# Practice and challenges of HB-HTA in China: insights from hospital management and clinical perspectives

**DOI:** 10.1017/S026646232610381X

**Published:** 2026-05-26

**Authors:** Yujie Xia, Sheng Han, Lanting Lyu

**Affiliations:** 1 Health Technology Assessment and Policy Evaluation Group, Renmin University of China, China; 2 International Research Center for Medicinal Administration, Peking University, China; 3 School of Population and Health, Renmin University of China, China

**Keywords:** hospital-based health technology assessment, hospital management, decision making, medical technology adoption, China

## Abstract

**Objectives:**

This study aims to investigate the awareness, practices, needs, and challenges of hospital-based health technology assessment (HB-HTA) in China’s tertiary public hospitals, drawing on perspectives from both hospital management and clinical practice.

**Methods:**

Semi-structured interviews were conducted with seventeen participants representing six major tertiary public hospitals in China. Respondents included vice presidents, clinical department directors, and heads of pharmacy. The questionnaire explored four dimensions consistent with China’s national performance appraisal framework: medical quality, operational efficiency, sustainable development, and satisfaction. Data were analyzed using NVivo 14 for qualitative responses and Excel 2021 for quantitative metrics.

**Results:**

The findings revealed that 82.35 percent (14/17) of hospitals lacked dedicated HB-HTA teams, with decisions made primarily by coordination between Medical Affairs Offices (28.89 percent, 13/45) and Pharmacy Departments (22.22 percent, 10/45). Safety (28.26 percent, 13/46), effectiveness (23.91 percent, 11/46), and cost-effectiveness (19.57 percent, 9/46) were prioritized criteria, yet only 29.41 percent (5/17) of institutions used formal HB-HTA tools. Divergence persisted between administrators’ organizational priorities and clinicians’ patient-centered perspectives. Despite barriers reported by 62.8 percent (27/43) of respondents’ institutions, 88.24 percent (15/17) expressed an urgent or recognized need for HB-HTA implementation.

**Conclusions:**

For effective HB-HTA implementation in China, addressing capacity deficits and reconciling stakeholder perspectives is essential. A project-based strategy, supported by government real-world data platforms and interdisciplinary teams, is recommended for contextually-appropriate evaluation frameworks.

## Introduction

Healthcare technology is experiencing rapid innovation, with short iteration cycles straining hospitals’ ability to allocate resources effectively. Tertiary hospitals in China now face over 100 new technology adoption requests annually (1), a surge that expands diagnostic and therapeutic capacity but complicates cost control and resource distribution. Systemic transformation is further driven by three developments: payment reforms in Basic Medical Insurance, rigorous management under public hospitals’ high-quality development initiatives, and a broader institutional shift toward enhanced regulatory oversight and decision-making accountability within the healthcare system. These forces highlight the need for scientific value-assessment frameworks that shift technology adoption from experience-based to evidence-based.

In response to these imperatives, China’s hospital-based health technology assessment (HB-HTA) evolved from localized explorations (2005–2017) to seven national pilot projects in 2018. These projects, none overlapping with this study’s sample, validated its utility in the assessments of surgical techniques, consumables, and management models (1;2). Concurrently, initiatives such as comprehensive clinical evaluation of pharmaceuticals and hospital-based assessments of high-value consumables transformed HB-HTA into an integrated decision-support mechanism spanning procurement, application, and management cycles (2).

However, persistent systemic challenges remain. Current HB-HTA practices are characterized by inconsistent methodologies, weakened scientific rigor, and limited application of findings (3;4). To address these gaps, this study adopts a three-pronged, qualitative approach centered on capturing stakeholders’ perspectives. First, we investigate technology assessment practices in tertiary public hospitals (TPHs) based on executives’ and clinicians’ accounts to identify deviations from standardized HB-HTA frameworks (e.g., the AdHopHTA guiding principles for good practices (5). Second, we explored their perceptions and attitudes toward HB-HTA, while identifying the priority evaluation dimensions (e.g., cost-effectiveness, clinical value, organizational impact, and patient perspectives) to inform future assessment design. Finally, we map perceived implementation barriers and propose context-specific strategies to establish sustainable HB-HTA models within China’s institutional landscape.

## Methods

### Questionnaire design

To address the research aims, a semi-structured questionnaire was developed by the research team, based on established policy frameworks and refined through pilot expert review, which comprised six open-ended and thirteen closed-ended items. Closed-ended questions were aligned with national benchmarks, including the *National Performance Appraisal Manual of Tertiary Public Hospitals (2023)* (6) and *Evaluation Indicators of High-Quality Development of Public Hospitals (2022)* (7). These frameworks ensure the survey addresses institutional priorities. And the *Notice on Enhancing Patient Experience of Medical Care (2023)* (8), a core national initiative establishing twenty-seven detailed indicators for patient-centric improvement.

The research team collected expert feedback on the initial questionnaire through an in-depth preliminary interview with an experienced director of pharmacy on 4 February 2024. Based on the feedback, our team held detailed discussions to refine the instrument. Revisions included three aspects: ① adding initially overlooked questions identified by the respondent; ② improving wording precision to reduce ambiguity; and ③ merging overlapping questions and options to enhance the clarity (see the Supplementary File S1 for the full questionnaire).

### Sampling strategy

Hospitals were selected via a two-stage, purposive sampling strategy. The study was initiated within a predefined pool of fifteen tertiary public hospitals (TPHs) accessible through our academic collaboration networks. Given the absence of a national HB-HTA registry, this network-based approach ensured practical access. From this pool, we first ensured the inclusion of hospitals from all of China’s Four Major Economic Regions (the Eastern, Central, Western, and Northeastern Regions) to achieve geographic diversity (9). Second, within each region, we prioritized TPHs with high national performance rankings or provincial prominence, as these leading institutions are most likely to possess substantive HB-HTA experience. This two-stage process resulted in the selection of six target hospitals for participant recruitment. In China’s healthcare system, “tertiary hospitals” represent the highest level and serve as primary referral centers, characterized by large scale, comprehensive specialties, and mandates for teaching and research.

From the six selected target hospitals, we identified a pool of thirty-two potential candidates, all of whom were senior leaders accessible through our academic collaboration networks. These candidates were physicians serving as clinical departmental directors or hospital executives – key agents in the multistage HB-HTA adoption pathway. Eligibility was assessed for all candidates. The key criterion, direct experience in HB-HTA, was defined to encompass both formal, structured activities and the informal decision-making processes embedded in committee-based hospital governance (i.e., technology adoption deliberations occurring within existing administrative hierarchies and the absence of a dedicated HTA body). Those who met all inclusion criteria were subsequently contacted via email or phone to schedule face-to-face interviews. While not statistically representative of all Chinese tertiary hospitals, this purposive sample is designed to yield profound insights from these information-rich cases.

### Interviewer training and quality control

Data were collected through in-person interviews at respondents’ workplaces. Before the interview, respondents received a paper copy of the questionnaire, whose first page contained a written overview detailing the study’s objectives and the research team. All interviews were conducted privately, with no nonparticipants present. To accommodate respondents’ wide geographic distribution of workplace, four interviewers carried out the fieldwork. All interviewers possessed at least two years of HTA research experience. All interviews were audio-recorded with prior verbal consent. Verbatim transcripts were produced by a research assistant; to further ensure consistency, these transcripts were then cross-checked against the participants’ corresponding questionnaire responses. Interviewers also maintained brief field notes to capture contextual observations and nonverbal cues. Before data collection, structured online training was implemented, focusing on: ① clarifying key HB-HTA terminologies; ② compiling a reference knowledge dossier comprising the AdHopHTA Handbook (5) and seven recent Chinese HB-HTA studies (2;10–15); and ③ standardizing interview protocols and probing techniques to minimize interviewer bias.

### Data analysis

For the open-ended survey questions, responses were analyzed using a conventional content analysis approach (16). Two researchers independently performed the analysis in NVivo 14. Guided by the research questions, the process involved open coding of transcripts, grouping codes into axial categories, and refining them into selective themes (17). This process summarized the perceptions, practices, needs, and challenges related to HB-HTA. For the closed-ended questions, Excel 2021 was used to calculate the frequency and proportion of each selected option (18;19), which identified the dimensions and metrics most applied and prioritized in assessments.

This study was conducted and reported in accordance with the Consolidated Criteria for Reporting Qualitative Research (COREQ) checklist (20) (see the Supplementary File S2).

## Results

### Overview of survey respondents

**Figure 1.**
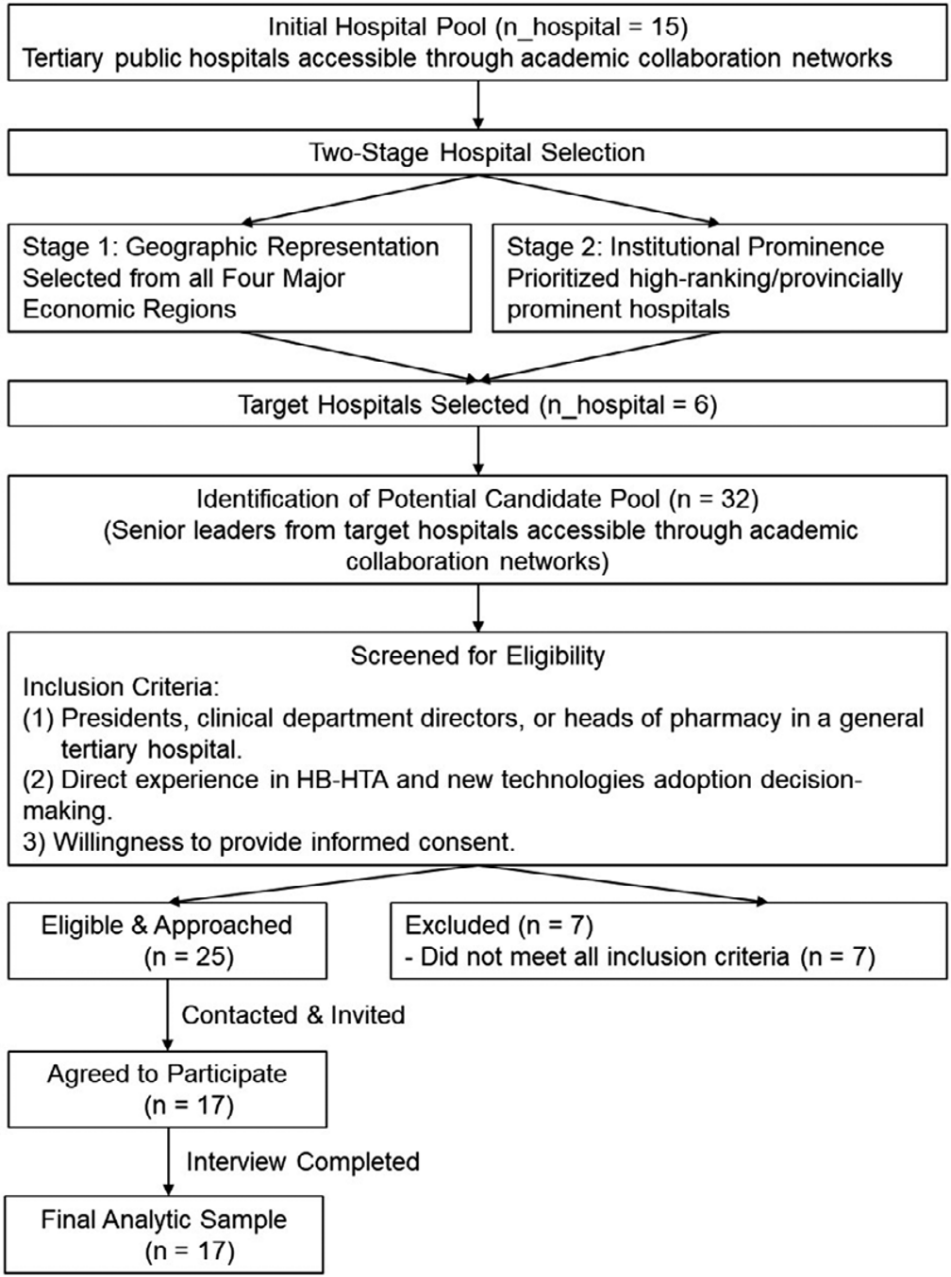
Flowchart of participant screening and enrollment. Figure 1. long description.

Among the 25 senior administrators and/or clinical leaders approached, 17 completed the interview, with a response rate of 68 percent. Scheduling conflicts were the primary reason for nonparticipation. The participant flow is detailed in Figure 1. These professionals were from tertiary hospitals with an average of 3,375 beds (range: 1,200–5,613), all of which served as major teaching and referral centers. Interviews averaged 45 minutes in duration (range: 35–60 minutes).

The seventeen participants were from six large-scale TPHs, including four comprehensive traditional Chinese medicine (TCM) hospitals that integrate TCM and modern medical services. This study focuses on the common HB-HTA processes for evaluating modern medical technologies. The respondents comprised four vice presidents, ten clinical department directors (from anesthesiology, neurosurgery, orthopedics, and gastrointestinal surgery), and three heads of the pharmacy department. All respondents had experience initiating or conducting new technology adoption assessments in their institutions (see Table 1).

**Table 1.** Demographic and professional characteristics of the survey respondents Table 1. long description.

	Number of respondents	Percentage (%)
**Type of hospital**		
General hospital	14	82.35%
Traditional Chinese Medicine hospital	3	17.65%
**Geographic region of the hospital**		
Eastern region	8	47.06%
Central region	5	29.41%
Western region	2	11.76%
Northeastern region	2	11.76%
**Respondent’s department**		
Office of the President	4	23.53%
Department of Anesthesiology	6	35.29%
Department of Pharmacy	3	17.65%
Department of Gastrointestinal Surgery	2	11.76%
Department of Orthopedics	1	5.88%
Department of Neurosurgery	1	5.88%
**Respondent’s title**		
Vice President	4	23.53%
Chief Physician	9	52.94%
Clinical Pharmacy Specialist	3	17.65%
Associate Chief Physician	1	5.88%

Given the qualitative nature and sample size of this study, descriptive statistics are presented as percentages followed by the actual number of occurrences and the base (n/N) to ensure transparency and avoid misinterpretation of generalizability.

### Awareness and current practices of HB-HTA

#### Stakeholders and processes in hospital new technology adoption

Decision-making involvement was most often reported for the Medical Affairs Office (28.89 percent, 13/45), followed by the Department of Pharmacy (22.22 percent, 10/45) and the Pharmaceutical Administration Committee (11.11 percent, 5/45) (see Table 2). The involvement of stakeholders from operations, pharmacy, ethics, insurance, and clinical sectors renders this decision-making process a multi-departmental collaborative effort. This ad-hoc, committee-based approach, which lacks a formalized HTA body and is deeply embedded within existing administrative hierarchies, aligns most closely with the “Internal Committee Model” as described by Cicchetti et al. (2018) (21). Approvals are granted through committees or ad-hoc meetings, with membership varying by technology type. Notably, 82.35 percent (14/17) of hospitals lacked dedicated HB-HTA teams or personnel. Only 17.65 percent (3/17) had designated committees or teams, and few possessed specialized HB-HTA expertise, which indicates continued reliance on administrative processes embedded in existing departmental duties rather than independent HB-HTA units.

**Table 2.** Identified themes regarding the perception, practice, needs, and challenges of HB-HTA Table 2. long description.

Themes and categories	Number of references	Percentage (%)
**1 Decision-making department on new technology adoption**	45	
1.1 Medical Affairs Office	13	28.89%
1.2 Department of Pharmacy	10	22.22%
1.3 Pharmaceutical Administration Committee	5	11.11%
1.4 Ethics Committee	4	8.89%
1.5 Related clinical departments	3	6.67%
1.6 Medical Insurance Office	3	6.67%
1.7 Equipment Office	2	4.44%
1.8 Pricing Office	2	4.44%
1.9 Operations Office	2	4.44%
1.10 Medical Education and Research Office	1	2.22%
**2 Is there a professional HB-HTA team?**	17	
2.1 No	14	82.35%
2.2 Yes	3	17.65%
**3 The most important factor for adoption**	46	
3.1 Safety	13	28.26%
3.2 Effectiveness	11	23.91%
3.3 Cost-effectiveness	9	19.57%
3.4 Clinical need	4	8.70%
3.5 Innovativeness	4	8.70%
3.6 Accessibility	3	6.52%
3.7 Operational impacts	2	4.35%
**4 Whether HB-HTA tools are used**	17	
4.1 No	10	58.82%
4.2 Yes	5	29.41%
4.3 Unknown	2	11.76%
**5 Current decision-making tools (non-HB-HTA)**	10	
5.1 Panel deliberation and collective voting	5	50.00%
5.2 Unknown	3	30.00%
5.3 Refer to clinical guidelines	1	10.00%
5.4 Refer to group standards	1	10.00%
**6 Perceived need for implementing HB-HTA**	17	
6.1 Urgent need	12	70.59%
6.2 Recognized need	3	17.65%
6.3 No perceived need	2	11.76%
**7 Reasons for requiring HB-HTA**	26	
**7.1 Promote evidence-based decision making**	14	53.85%
7.1.1 Establish clear decision-making criteria	4	15.38%
7.1.2 Ensure comprehensive technology assessments	2	7.69%
7.1.3 Create a transparent decision-making process	1	3.85%
7.1.4 Monitor long-term safety and effectiveness post-market	1	3.85%
**7.2 Support hospital management and operations**	7	26.92%
7.2.1 Enable dynamic technology life cycle management	1	3.85%
7.2.2 Keep pace with rapid technological innovation	1	3.85%
7.2.3 Optimize resource allocation	1	3.85%
7.2.4 Fulfill performance evaluation criteria	1	3.85%
7.2.5 Prevent conflicts of interest	1	3.85%
**7.3 Improve the quality of medical care**	5	19.23%
7.3.1 Enhance clinical staff proficiency with new technology	2	7.69%
7.3.2 Ensure patient safety	1	3.85%
7.3.3 Better address patient care needs	1	3.85%
**8 Challenges in implementing HB-HTA**	43	
**8.1 Institutional and resource barriers**	27	62.79%
8.1.1 Shortage of interdisciplinary expertise	9	20.93%
8.1.2 Lack of high-quality data	6	13.95%
8.1.3 Insufficient data availability	5	11.63%
8.1.4 Lack of leadership support	4	9.30%
8.1.5 Unclear organizational structure or reporting guidelines	2	4.65%
8.1.6 Absence of a quality control framework	1	2.33%
**8.2 Methodological challenges**	12	27.91%
8.2.1 Requirements for sophisticated and rigorous methodologies	6	13.95%
8.2.2 Challenges in adapting evaluations to multiple clinical contexts	4	9.30%
8.2.3 Difficulties in establishing clear goals and reaching stakeholders’ consensus	2	4.65%
**8.3 Systemic and procedural barriers**	4	9.30%
8.3.1 Lack of systematic collaboration and coordination	2	4.65%
8.3.2 Resource and capability constraints for implementing recommendations	2	4.65%
**9 Are you willing to use the HB-HTA decision model?**	17	
9.1 Yes	17	100.00%

*Note:* HB-HTA, hospital-based health technology assessment.

#### Application of HTA in hospital decision making

A clear gap persists between recognition and application of HTA in China’s tertiary hospitals. Although decision makers acknowledge core HTA dimensions during new technology adoption, the systematic use of formal HTA tools remains limited. Safety (28.26 percent, 13/46), effectiveness (23.91 percent, 11/46), and cost-effectiveness (19.57 percent, 9/46) were the most valued dimensions in technology adoption approval, indicating that decision-makers’ attention aligns with core HTA concepts and a prioritization of clinical and economic criteria. This contrasts with the under-representation of organizational and operational impacts (e.g., staff training and performance metrics), which received minimal attention. This pattern indicates that current assessments do not fully align with comprehensive HB-HTA frameworks, such as AdHopHTA (5), which explicitly include organizational dimensions alongside clinical and economic evaluations. Only 29.41 percent (5/17) of hospitals reported using dedicated HB-HTA tools. Among these, comprehensive drug evaluation frameworks were the most applied (60.00 percent, 3/5). These frameworks, while guided by a national policy (22), represent diverse local adaptations rather than a unified tool. By contrast, a lack of structured HTA tool application was reported by 58.82 percent (10/17) of the respondents. In these cases, decisions relied mainly on panel deliberation and collective expert voting (50.00 percent, 5/10), as well as on clinical guidelines or group standards (10.00 percent each, 1/10), especially within TCM institutions. These findings highlight a clear disconnect: while there is broad conceptual endorsement of HTA principles among hospital leaders, this has not been operationalized into the routine use of systematic methodologies for decision making. This indicates a more fundamental deficit: the recognized importance of HTA dimensions has not translated into their institutionalization through structured tools and processes.

### Motivations, challenges, and future adoption

#### Perceived urgency and motivations for implementing HB-HTA

An overwhelming majority of respondents expressed a strong demand for establishing or strengthening HB-HTA in their institutions. The survey results showed that 70.59 percent (12/17) of the respondents considered implementing HB-HTA in their institutions as “urgent need,” while another 17.65 percent (3/17) reported a “recognized need,” yielding a combined 88.24 percent (15/17) in favor. Only 11.76 percent (2/17) regarded it as “no perceived need,” due to concerns about administrative burden or a preference for relying on published evidence. The leading motivator, “promoting evidence-based decision-making” (53.85 percent, 14/26), reflects the need to establish clear and transparent evaluation mechanisms to address current limitations such as decentralized decision making, subjective judgments, and ethical risks. Additional drivers included “support hospital management and operations” (26.92 percent, 7/26) and “improve the quality of medical care” (19.23 percent, 5/26). This demand is further driven by rapid medical innovation, constrained financial and human resources, and increased scrutiny of procurement decisions, necessitating more transparent decision-making processes.

#### Key challenges in HB-HTA implementation

According to our survey, the implementation of HB-HTA faces challenges that can be grouped into three categories: institutional and resource barriers, methodological challenges, and systemic and procedural barriers. The most frequently cited barrier was institutional and resource barriers (62.79 percent, 27/43), encompassing a shortage of interdisciplinary expertise (20.93 percent, 9/43), lack of high-quality data (13.95 percent, 6/43), and insufficient data availability (11.63 percent, 5/43). Lack of leadership support (9.30 percent, 4/43) was also identified as a critical obstacle. Respondents with prior experience in new technology adoption were more receptive to HB-HTA, whereas those without experience often perceived it as a bureaucratic burden. Methodological challenges (27.91 percent, 12/43) presented another obstacle, reflected in concerns about sophisticated and rigorous methodologies (13.95 percent, 6/43), adapting evaluations to multiple clinical contexts (9.30 percent, 4/43), and establishing clear goals and reaching stakeholders’ consensus (4.65 percent, 2/43). Implementation was further complicated by defining evaluation priorities (such as social benefit, research advancement, or economic impact) and balancing perspectives across clinical departments and hospital leadership. Systemic and procedural barriers (9.30 percent, 4/43) also hindered adoption. Among institutions with HB-HTA experience, small teams and excessive workloads restricted both the scope and depth of the evaluation.

#### Willingness to adopt HB-HTA tools

Despite these challenges, all respondents (100 percent, 17/17) expressed willingness to adopt an HB-HTA decision-making model or digital platform. Key factors influencing this willingness included cost-effectiveness, procedural simplicity, scientific rigor, resource sharing, and contextual adaptability. Respondents also stressed the importance of avoiding redundant competition among various platforms.

### Dimensions and indicators in HB-HTA frameworks

This section reports results on the four predefined dimensions of the HB-HTA framework, which were quantified from the closed-ended survey questions.

#### Medical quality

**Figure 2.**
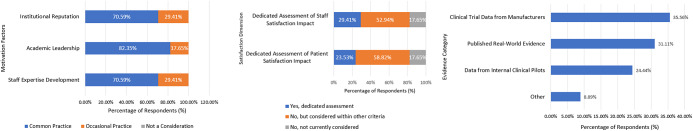
Key factors in new technology adoption assessment at hospitals. (a) Motivations for adoption based on sustainable development factors. (b) Formal assessment of satisfaction impacts. (c) Primary types of evidence used for safety and efficacy evaluation. Figure 2. long description.

In HB-HTA, evaluations of safety and effectiveness are well-established, and the metrics vary by technology. This study examined the evidence sources used for these assessments. The findings revealed that China’s TPHs rely primarily on external evidence when evaluating new health technologies, reflecting limited capacity to generate internal data. Clinical trial data from manufacturers were the most common source (35.56 percent, 16/45), followed by real-world data from the literature (31.11 percent, 14/45). Only 24.44 percent (11/45) of the respondents reported the use of internal trial data, and 8.89 percent (4/45) cited expert consensus or guidelines (see Figure 2). Despite regulatory efforts in China to integrate real-world evidence into practice, hospital-generated data remain underutilized.

#### Operational efficiency

The prevailing practice emphasizes direct, visible costs over systemic or indirect costs, which limits the analytical depth of the evaluations. Direct medical costs (36.59 percent, 15/41) and performance metrics (36.59 percent, 15/41) were the most frequently assessed elements (see Table 3). Direct costs included insurance reimbursement (27.78 percent, 15/54), drug and consumable costs (25.93 percent, 14/54), and medical service fees (18.52 percent, 10/54). Common performance indicators included cost per admission increase (21.74 percent, 15/69), inpatient essential drug use (20.29 percent, 14/69), and volume-based procurement drug use (20.29 percent, 14/69). Only 24.39 percent (10/41) of institutions accounted for operational costs, with the top three items (supporting equipment, labor, and storage) each considered by 25 percent (10/40) of the respondents, and training by 22.5 percent (9/40). Among those conducting cost-effectiveness analyses, 47.06 percent (8/17) considered only direct medical costs, overlooking broader organizational implications and diverging from a hospital-based perspective. These findings highlight two shortcomings in current HB-HTA practice: the underestimation of hidden outlays and operational impacts, and the lack of long-term outcome tracking, which diminishes its strategic value.

**Table 3.** Operational efficiency in hospital technology adoption: assessed dimensions Table 3. long description.

Primary category	Specific aspect	Percentage (%)	
**A. Direct medical cost**		**36.59%**	
	A.1 Insurance reimbursement		27.78%
	A.2 Drug and consumable costs		25.93%
	A.3 Medical service fees		18.52%
	A.4 Adverse event management costs		14.81%
	A.5 Length of hospital stay		12.96%
**B. Performance metrics**		**36.59%**	
	B.1 Cost per admission increase		21.74%
	B.2 Volume-based procurement drug use		20.29%
	B.3 Inpatient essential drug use		20.29%
	B.4 Medical service revenue		17.65%
	B.5 High-value consumable revenue		17.65%
	B.6 Other		1.47%
**C. Operational cost**		**24.39%**	
	C.1 Labor		25.00%
	C.2 Storage		25.00%
	C.3 Supporting equipment		25.00%
	C.4 Training		22.50%
	C.5 Other		2.50%
**D. Other**		**2.44%**	

#### Sustainable development

The potential of new technologies to support disciplinary growth, institutional reputation, and staff expertise is a major factor influencing HB-HTA adoption decisions. The survey revealed that 82.35 percent (14/17) of institutions considered adopting technologies that strengthen academic leadership and disciplinary development, while 70.59 percent (12/17) prioritized those that improve staff expertise or enhance institutional branding (see Figure 2). This pattern reflects a strong orientation toward academic values in China’s TPHs – a focus that is consistent with the professional title system, which rewards research output and publications.

#### Patient and staff satisfaction

The assessment of both patient and staff satisfaction is indirect and inconsistent during hospital technology assessments, and neither is typically treated as an independent dimension. Only 23.53 percent (4/17) of hospitals were found to use a dedicated process to evaluate patient satisfaction, and 29.41 percent (5/17) applied a comparable process for staff satisfaction. Most institutions integrated satisfaction into other assessment dimensions (58.82 percent, 10/17 for patients, 52.94 percent, 9/17 for staff; see Figure 2). These practices are rarely documented in final reports and seldom influence adoption decisions, demonstrating that both patient and staff satisfaction are subordinate to technical metrics and carry limited weight in the evaluation process.

## Discussion

### Systemic gaps and impetus for HB-HTA

Survey results indicate that HB-HTA implementation in China’s tertiary hospitals faces a critical gap between recognized urgency and systemic capacity. This is reflected in a fundamental disconnect: a high awareness and valuation of core HTA dimensions coexist with a stark lack of institutionalized, systematic methodologies for their application. This gap persists due to capacity constraints, lack of formal structures, and reliance on external evidence, which collectively compromise the reliability and narrow the scope of hospital-level assessments. This challenge is reflected in the dominance of committee-based assessments in our sample, which contrasts with the more established independent HTA units reported in leading hospitals globally (23), indicating an earlier stage of formalization. And the severe shortage of dedicated personnel (noted by 82.35 percent of respondents) constitutes a near-universal challenge, strongly echoed in international studies (23–25).

This gap is further reflected in the limited use of formal HTA tools despite the prioritization of core evaluation criteria. The transition from awareness to practice is complicated by the need for contextually adapted methodologies that balance both clinical and managerial perspectives, along with a reliance on external evidence. Given these systemic challenges, a government-led platform is crucial to provide the necessary methodologies, data infrastructure, and coordination, thereby enhancing internal capacity across institutions. This need for a coordinated approach is reflected globally (23;25).

Furthermore, a strong impetus for HB-HTA is driven by a systemic shift toward procedural decision making to mitigate risks under new payment models and performance evaluations. China’s context introduces unique drivers, such as the DRG/DIP payment reforms and public hospital performance appraisals, which are less prominent in the international literature. This comparison underscores that while foundational barriers are shared, the evolution of HB-HTA is deeply shaped by the local healthcare policy ecosystem.

### Divergent perspectives on HB-HTA: hospital administrators versus clinical experts

Substantial differences exist between administrators and clinicians in their perceptions of HB-HTA, reflecting different priorities, approaches, and mindsets. Administrators emphasized organizational efficiency, cost control under the Basic Medical Insurance System, disciplinary growth, and institutional reputation. They viewed new technologies as strategic instruments for academic advancement and competitive positioning, and valued structured, explicit patient evaluations of satisfaction. For instance, when discussing the motivation for adopting new technologies, one vice president stated, “We must consider how this technology will elevate our hospital’s academic profile and help us compete in regional rankings.” (Participant #12). Another administrator emphasized the need for technologies that “fit into the long-term strategic plan of our institution, not just solve an immediate clinical problem” (Participant #04). Clinicians, by contrast, prioritized clinical value, focusing on safety, effectiveness, and patients’ immediate needs. Their support for technology was demand-driven and evidence-based. As one neurosurgeon explained, “My primary concern is always, do no harm. Then, does it truly improve patient outcomes compared to what we already do?” (Participant #09). They preferred an informal approach to assessing patient satisfaction, relying on professional judgment rather than formal metrics. Furthermore, administrators demonstrated strong interest in applying HB-HTA for resource allocation and supporting standardized tools to enhance efficiency, yet faced barriers such as fragmented data and staffing shortages, as data show above. Clinicians were more cautious owing to concerns about additional administrative burden and misalignment with clinical practice; they therefore emphasized the need for flexibility and context-specific adaptation. Economically, administrators promoted comprehensive business-case analyses that capture operational and performance impacts on the hospital budget and expenditures, whereas clinicians concentrated on direct costs such as drugs, consumables, and reimbursement.

These differences indicate distinct cognitive frameworks: administrators are typically characterized by a systematic mindset, focusing on long-term strategy, while clinicians are guided by a situational orientation, prioritizing immediate patient needs. The study also found that clinicians with prior experience in successfully introducing new technologies tended to adopt more open and positive attitudes toward HB-HTA tools, while those without such experience were more likely to view them as “administrative shackles.” Bridging these perspectives is critical to the effective implementation of HB-HTA in China’s tertiary hospitals.

### Promoting HB-HTA in China: a project-based approach and platform building

The survey confirms strong demand for HB-HTA within China’s TPHs, given its wide recognition as an evidence-based decision-making tool. We accordingly discuss two potential primary implementation pathways.

The first pathway involves complete integration into hospital management structures, which entails institutionalizing procedures and designating specialized staff. This centralized model aligns with conventional advisory frameworks but faces significant challenges, as mentioned before. By contrast, a project-based approach offers a more adaptable alternative by focusing on specific technologies such as innovative devices or high-cost consumables. This model could enhance hospital engagement by delivering tangible, short-term gains in clinical and operational outcomes, directly addressing the lack of “perceived benefit” noted in the international literature (26). It also improves resource efficiency and is more responsive to context-specific needs. Critically, this approach could be more acceptable in hospitals with traditional management styles, as it avoids immediate systemic overhaul and helps alleviate concerns about cost control under DRG/DIP payment reforms.

The project-based model, while reducing reliance on internal HTA agencies, highlights the need for inter-institutional platforms. Such platforms are essential for aggregating real-world data, countering fragmented evaluations, minimizing redundancy, and promoting collective learning. However, the implementation of such platforms faces significant challenges, as data sharing is often hindered by hospitals’ perceived risk of exposing sensitive information and a lack of interoperable data standards. Therefore, this reality accentuates the indispensable role of proactive government leadership in building trust mechanisms, establishing robust data governance frameworks, and creating clear incentives for participation.

Beyond hospital-level applications, HB-HTA holds broader strategic value. Establishing long-term monitoring of reimbursed drugs through real-world data and HB-HTA can address evidence gaps from randomized controlled trials (e.g., long-term outcomes and subgroup populations). This dynamic process supports evidence-based updates to the reimbursement catalog, thereby steering insurance resources toward higher-value technologies and linking hospital-level assessments with national HTA systems. From 2025, the active promotion of real-world study by national health sectors provides a pivotal top-down driver to address the challenges of data sharing and platform building in the project-based model (27).

### Strengths and limitations

This study demonstrates three principal strengths. First, it captures a critical phase in the development of China’s HB-HTA. Following its introduction in national policy in 2018, HB-HTA entered a pilot stage, during which related practices increased markedly. However, this momentum was partly interrupted by the COVID-19 pandemic. Our study systematically examines the post-pandemic implementation of HB-HTA in TPHs across China, extending previous policy tracking and reflecting new realities. Second, methodologically, it incorporates the dual perspectives of hospital administrators and clinicians, ensuring authoritative and multidimensional insights. Third, the study proposes a forward-looking strategy grounded in real-world evidence, emphasizing project-driven initiatives and platform construction, which provides actionable suggestions for promoting HB-HTA within China’s hospital system.

This study has three limitations. First, the sample size is limited due to the difficulty of recruiting senior administrators from TPHs. However, our internal expert panel determined that thematic saturation had been achieved, as new themes and perspectives rarely emerged in the later stages of the survey. Second, although the sample hospitals are drawn from all the Four Major Economic Regions, most are concentrated in the Eastern and Central parts of the country, with an underrepresentation of hospitals from the Western, Northeastern, and border regions. Thus, the distinct perceptions and practices of hospitals in these areas may not be fully captured. Third, the qualitative nature of this study may involve social desirability bias and framing effects from interview prompts.

## Conclusion

This study reveals three key findings on HB-HTA implementation in China’s TPHs. First, administrators and clinicians diverge systematically in priorities and methods. Second, operational gaps, including the widespread lack of dedicated HB-HTA teams, an overemphasis on direct costs, and the underuse of satisfaction metrics, hinder standardization. Third, project-based pathways appear more feasible than institutional reform, improving resource efficiency and adaptability.

To advance HB-HTA, we recommend: establishing government-led platforms to standardize methodologies and share learning; forming interdisciplinary hospital teams that integrate clinical and economic expertise; and implementing modular HB-HTA tools that balance standardization with contextual flexibility.

## Supporting information

10.1017/S026646232610381X.sm001 Xia et al. supplementary material 1 Xia et al. supplementary material

10.1017/S026646232610381X.sm002 Xia et al. supplementary material 2 Xia et al. supplementary material

## Supplementary material

The supplementary material for this article can be found at http://doi.org/10.1017/S026646232610381X.

## Long descriptions

### Figure 1. Long description

At the top, the initial hospital pool is defined as 15 tertiary public hospitals accessible via academic collaboration networks. An arrow leads down to two-stage hospital selection. The first stage, on the left, is geographic representation, selecting from all four major economic regions. The second stage, on the right, is institutional prominence, prioritizing high-ranking or provincially prominent hospitals. Both stages converge to target hospitals selected, totaling 6. Below, identification of a potential candidate pool (n = 32) is described as senior leaders from target hospitals accessible through academic collaboration networks. Next, screening for eligibility is based on three criteria: being a president, clinical department director, or head of pharmacy in a general tertiary hospital; direct experience in H B dash H T A and new technology adoption decision-making; and willingness to provide informed consent. The flow splits: on the left, 25 eligible and approached, and on the right, 7 excluded for not meeting all inclusion criteria. The eligible group is contacted and invited, with 17 agreeing to participate. The process continues downward to interview completion and ends with the final analytic sample of 17.

Navigate back to Figure 1..

### Table 1. Long description

The table is organized into four main sections. First, type of hospital: General hospital 14 respondents 82.35 percent, Traditional Chinese Medicine hospital 3 respondents 17.65 percent. Next, geographic region: Eastern region 8 respondents 47.06 percent, Central region 5 respondents 29.41 percent, Western region 2 respondents 11.76 percent, Northeastern region 2 respondents 11.76 percent. Third, respondent’s department: Office of the President 4 respondents 23.53 percent, Department of Anesthesiology 6 respondents 35.29 percent, Department of Pharmacy 3 respondents 17.65 percent, Department of Gastrointestinal Surgery 2 respondents 11.76 percent, Department of Orthopedics 1 respondent 5.88 percent, Department of Neurosurgery 1 respondent 5.88 percent. Last, respondent’s title: Vice President 4 respondents 23.53 percent, Attending Physician 9 respondents 52.94 percent, Clinical Pharmacy Specialist 3 respondents 17.65 percent, Associate Attending Physician 1 respondent 5.88 percent.

Navigate back to Table 1..

### Table 2. Long description

From the top, the table lists nine main themes regarding HB-HTA, each with subcategories. Theme 1, Decision-making department on new technology adoption, has 45 references and ten subcategories: Medical Affairs Office (13, 28.89 percent), Department of Pharmacy (10, 22.22 percent), Pharmaceutical Administration Committee (5, 11.11 percent), Ethics Committee (4, 8.89 percent), Related clinical departments (3, 6.67 percent), Medical Insurance Office (3, 6.67 percent), Equipment Office (2, 4.44 percent), Pricing Office (2, 4.44 percent), Operations Office (2, 4.44 percent), Medical Education and Research Office (1, 2.22 percent). Theme 2, Is there a professional HB-HTA team, has 17 references: No (14, 82.35 percent), Yes (3, 17.65 percent). Theme 3, The most important factor for adoption, has 46 references: Safety (13, 28.26 percent), Effectiveness (11, 23.91 percent), Cost-effectiveness (9, 19.57 percent), Clinical need (4, 8.70 percent), Innovativeness (4, 8.70 percent), Accessibility (3, 6.52 percent), Operational impacts (2, 4.35 percent). Theme 4, Whether HB-HTA tools are used, has 17 references: No (10, 58.82 percent), Yes (5, 29.41 percent), Unknown (2, 11.76 percent). Theme 5, Current decision-making tools (non-HB-HTA), has 10 references: Panel deliberation and collective voting (5, 50.00 percent), Unknown (3, 30.00 percent), Refer to clinical guidelines (1, 10.00 percent), Refer to group standards (1, 10.00 percent). Theme 6, Perceived need for implementing HB-HTA, has 17 references: Urgent need (12, 70.59 percent), Recognized need (3, 17.65 percent), No perceived need (2, 11.76 percent). Theme 7, Reasons for requiring HB-HTA, has 26 references and three subthemes: Promote evidence-based decision making (14, 53.85 percent) with four subcategories—Establish clear decision-making criteria (4, 15.38 percent), Ensure comprehensive technology assessments (2, 7.69 percent), Create a transparent decision-making process (1, 3.85 percent), Monitor long-term safety and effectiveness post-market (1, 3.85 percent); Support hospital management and operations (7, 26.92 percent) with five subcategories—Enable dynamic technology life cycle management (1, 3.85 percent), Keep pace with rapid technological innovation (1, 3.85 percent), Optimize resource allocation (1, 3.85 percent), Fulfill performance evaluation criteria (1, 3.85 percent), Prevent conflicts of interest (1, 3.85 percent); Improve the quality of medical care (5, 19.23 percent) with three subcategories—Enhance clinical staff proficiency with new technology (2, 7.69 percent), Ensure patient safety (1, 3.85 percent), Better address patient care needs (1, 3.85 percent). Theme 8, Challenges in implementing HB-HTA, has 43 references and three subthemes: Institutional and resource barriers (27, 62.79 percent) with six subcategories—Shortage of interdisciplinary expertise (9, 20.93 percent), Lack of high-quality data (6, 13.95 percent), Insufficient data availability (5, 11.63 percent), Lack of leadership support (4, 9.30 percent), Unclear organizational structure or reporting guidelines (2, 4.65 percent), Absence of a quality control framework (1, 2.33 percent); Methodological challenges (12, 27.91 percent) with three subcategories—Requirements for sophisticated and rigorous methodologies (6, 13.95 percent), Challenges in adapting evaluations to multiple clinical contexts (4, 9.30 percent), Difficulties in establishing clear goals and reaching stakeholders’ consensus (2, 4.65 percent); Systemic and procedural barriers (4, 9.30 percent) with two subcategories—Lack of systematic collaboration and coordination (2, 4.65 percent), Resource and capability constraints for implementing recommendations (2, 4.65 percent). Theme 9, Are you willing to use the HB-HTA decision model, has 17 references: Yes (17, 100.00 percent).

Navigate back to Table 2..

### Figure 2. Long description

Panel A, at the left, shows three grouped horizontal bars for motivation factors: Institutional Reputation, Academic Leadership, and Staff Expertise Development. Each bar is divided into blue for Common Practice, orange for Occasional Practice, and gray for Not a Consideration. Academic Leadership has the highest Common Practice at 82.35 percent, while Institutional Reputation and Staff Expertise Development both have 70.59 percent Common Practice and 29.41 percent Occasional Practice. Panel B, in the center, displays two grouped bars for satisfaction information: Dedicated Assessment of Staff Satisfaction Impact and Dedicated Assessment of Patient Satisfaction Impact. Each bar is split into blue for Yes, dedicated assessment, orange for No, but considered within other criteria, and gray for No, not currently considered. Patient Satisfaction Impact has 58.82 percent Yes, dedicated assessment, while Staff Satisfaction Impact has 29.41 percent Yes, dedicated assessment and 52.94 percent No, but considered within other criteria. Panel C, at the right, presents four horizontal bars for evidence categories: Clinical Trial Data from Manufacturers at 35.56 percent, Published Real-World Evidence at 31.11 percent, Data from Internal Clinical Pilots at 24.44 percent, and Other at 8.89 percent. All panels use the x-axis for Percentage of Respondents.

Navigate back to Figure 2..

### Table 3. Long description

The table is organized into four primary categories listed vertically: A. Direct medical cost at 36.59 percent, B. Performance metrics at 36.59 percent, C. Operational cost at 24.39 percent, and D. Other at 2.44 percent. Under A. Direct medical cost, the specific aspects and their percentages are: A.1 Insurance reimbursement 27.78 percent, A.2 Drug and consumable costs 25.93 percent, A.3 Medical service fees 18.52 percent, A.4 Adverse event management costs 14.81 percent, A.5 Length of hospital stay 12.96 percent, and A.6 Other 0.00 percent. Under B. Performance metrics: B.1 Cost per admission increase 21.74 percent, B.2 Volume-based procurement drug use 20.29 percent, B.3 Inpatient essential drug use 20.29 percent, B.4 Medical service revenue 17.65 percent, B.5 High-value consumable revenue 17.65 percent, and B.6 Other 1.47 percent. Under C. Operational cost: C.1 Labor 25.00 percent, C.2 Storage 25.00 percent, C.3 Supporting equipment 25.00 percent, C.4 Training 22.50 percent, and C.5 Other 2.50 percent. D. Other is listed as a single entry at 2.44 percent.

Navigate back to Table 3..
