# Supplementary material for: Practice and challenges of HB-HTA in China: insights from hospital management and clinical perspectives
Source: Int J Technol Assess Health Care. 2026 May 26;42(1):e57. doi: 10.1017/S026646232610381X (PMC13319477; doi:10.1017/S026646232610381X)
Supplement: Xia et al. supplementary material 2 — Xia et al. supplementary material [file S026646232610381Xsup002.docx]

**Questionnaire on the Current Hospital-Based Health Technology Assessment (HB-HTA) in China's Tertiary Public Hospitals**

Dear Expert,

Thank you for participating in this survey. As China continues to reform its payment method for Basic Medical Insurance and emphasizes high-quality development of public hospitals, the evidence-based adoption of new medical technologies has become an urgent priority. Medical technologies encompass a wide range of products and processes, including pharmaceuticals, consumables, instruments, equipment, clinical pathways, and medical services. In practice, Chinese hospitals generally classify these into two access pathways: new drugs and new technologies (the latter typically including consumables, instruments, and equipment).

HB-HTA provides evidence-informed decision-making support from the hospital perspective. It employs standardized processes to evaluate safety, effectiveness, economic factors, and organizational impacts (both internal and external). However, HB-HTA practice in China is still nascent, with limited standardization, uneven implementation, and modest influence on final decisions.

This survey aims to identify the key concerns and indicators that hospital administrators and clinicians prioritize during HB-HTA. The findings will contribute to developing a practical, clinically informed decision-making model for new technology access in China’s hospitals.

The questionnaire is structured around the framework from the *Performance Appraisal Manual of National Tertiary Public Hospitals (2023)*, covering medical quality, operational efficiency, sustainable development, and satisfaction. It also incorporates insights from recent policies such as the *Evaluation Indicators of High-Quality Development of Public Hospitals (2022)* and the *Notice on Enhancing Patient Experience of Medical Care (2023)*, as well as international protocols like the *AdHopHTA Handbook*.

All data collected in this survey will be used solely and strictly for academic research.

**I. Open-Ended Questions**

1. Which departments in your institution are responsible for decision-making regarding access to new drugs and technologies? Is there a professional HB-HTA team or personnel among them?
2. What are the most important factors considered by your institution when evaluating new drugs and technologies?
3. Does your institution utilize HB-HTA tools when considering access to new drugs and technologies? If not, what decision-making methods are currently used, and how effective are they?
4. What is the urgency of the need for your institution to adopt HB-HTA tools to enhance evidence-based decision-making? Why?
5. What potential difficulties might your institution face in conducting HB-HTA?
6. Would your institution consider adopting a comprehensive HB-HTA decision model with a web-based platform for inputting and analyzing data?

**II. Closed-Ended Questions**

- **Medical Quality**

1. What is the primary source of evidence your institution uses to evaluate the safety and effectiveness of new drugs and technologies? ( )

A. Clinical trial data provided by originator enterprises

B. Real-world data from the literature

C. Data generated from trial use within your institution

D. Other (Please specify): _____________________________

- **Operational Efficiency**

1. What are the key economic factors your institution evaluates when considering the introduction of new drugs and technologies? ( )

A. Direct medical costs

B. Indicators under the *Performance Appraisal Framework of National Tertiary Public Hospitals*

C. Operational costs at the organizational level

D. Other (Please specify): _____________________________

1. What are the aspects of your institution's evaluation regarding the impact of new drugs or technologies on direct medical costs? ( ) [Please answer this question only if you selected "A" in Question 2]

A. Whether covered by Basic Medical Insurance

B. Drug and consumable costs

C. Medical service fees

D. Management costs for adverse events

E. Length of hospital stay

F. Other (Please specify): _____________________________

1. Which key indicators under the *Performance Appraisal Framework of National Tertiary Public Hospitals* does your institution assess for effects resulting from new drugs or technologies? ( ) [Please answer this question only if you selected B in Question 2]

A. Inpatient basic drug utilization rate and procurement variety percentage

B. Utilization rate of pooled-procurement medicines

C. Proportion of medical service revenue to total medical revenue

D. Growth in average cost per hospitalization and associated drug costs

E. Revenue share of high-value medical consumables

F. Other (Please specify): _____________________________

1. Which operational cost categories does your institution analyze when new drugs or technologies are introduced? ( ) [Please answer this question if you selected C in Question 2]

A. Human resource costs

B. Necessary training required

C. Storage and maintenance costs

D. Supporting instruments and equipment

E. Other (Please specify): _____________________________

1. Does your institution conduct cost-effectiveness analyses? ( )

A. Yes, direct medical cost-effectiveness

B. Yes, operational cost-effectiveness

C. Yes, comprehensive cost-effectiveness (both direct and operational)

D. No, cost-effectiveness analysis is not conducted because: __________________

- **Sustainable Development**

1. How frequently does the potential to enhance staff expertise influence your institution's decision to adopt new drugs or technologies? ( )

A. Often

B. Sometimes

C. Never

1. How frequently does the potential to achieve academic leadership and advance the discipline influence your institution's decision to adopt new drugs or technologies? ( )

A. Often

B. Sometimes

C. Never

1. How frequently does the potential to enhance the institution's reputation and brand image influence your institution's decision to adopt new drugs or technologies? ( )

A. Often

B. Sometimes

C. Never

- **Satisfaction**

1. Is the prospective impact on patient satisfaction a formal evaluation criterion for new drug and technology adoption? ( )

A. Yes, it is a formal criterion

B. It is considered, but not as a formal criterion

C. No, it is not considered

1. What is the basis for judgment when your institution considers the potential impact of new drugs and technologies on patient satisfaction? ( ) [If you selected C in Question 10, please skip this question.]

A. Safety

B. Effectiveness

C. Procedural convenience

D. Treatment costs

E. Other (please specify): ________________

1. Is the prospective impact on medical staff satisfaction a formal evaluation criterion for new drug and technology adoption? ( )

A. Yes, it is a formal criterion

B. It is considered, but not as a formal criterion

C. No, it is not considered

1. What is the basis for judgment when your institution considers the potential impact of new drugs and technologies on medical staff satisfaction? ( ) [If you selected C in Question 12, please skip this question.]

A. Workload and procedural complexity

B. Treatment time and workflow efficiency

C. Difficulty of patient prognosis management

D. Medical staff remuneration and incentives

E. Other (please specify): ________________

**III. Questionnaire Feedback**

We value your feedback. Please share any suggestions or topics we may have missed to help us improve this questionnaire.
